# Supplementary material for: Genetic and Pharmacological Inhibition of Autophagy Increases the Monoubiquitination of Non-Photosynthetic Phosphoenolpyruvate Carboxylase
Source: Plants (Basel). 2020 Dec 23;10(1):12. doi: 10.3390/plants10010012 (PMC7823769; doi:10.3390/plants10010012)
Supplement: Supplementary file 1 [file plants-10-00012-s001.pdf]

Supplementary Fig. S1. Mascot peptide mass fingerprint analysis of the p110 band from tobacco cultured cells extract.

Mascot Search Results

User :
Email :
Search title :
MS data file : DATA.TXT
Database : Nicotiana-PEPCs 230719 (11 sequences; 8949 residues)
Timestamp : 3 Oct 2019 at 11:06:11 GMT
Top Score : 25 for Niben101Scf00212g03010.1, sequence match in blast db N.benthamiana Genome v1.0.1 predicted proteins PPC3-L4

Mascot Score Histogram

Protein score is -10*Log(P), where P is the probability that the observed match is a random event.
Protein scores greater than 23 are significant (p<0.05).

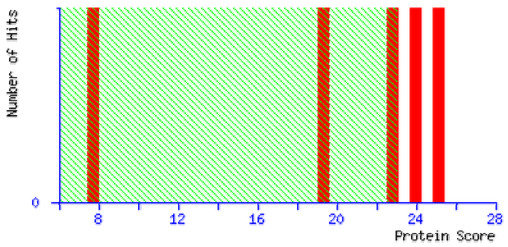

Concise Protein Summary Report

Format As | Concise Protein Summary | Help

Significance threshold p< 0.05 Max. number of hits 20

Re-Search All | Search Unmatched

1. Niben101Scf00212g03010.1 **Mass:** 116969 **Score:** 25 **Expect:** 0.034 **Matches:** 9
sequence match in blast db N.benthamiana Genome v1.0.1 predicted proteins PPC3-L4

---

2. Niben101Scf04036g04008.1 **Mass:** 109934 **Score:** 24 **Expect:** 0.048 **Matches:** 10
sequence match in blast db N.benthamiana Genome v1.0.1 predicted proteins PPC3-L2
Niben101Scf03439g03004.1 **Mass:** 109825 **Score:** 19 **Expect:** 0.13 **Matches:** 8
sequence match in blast db N.benthamiana Genome v1.0.1 predicted proteins PPC3-L3

---

3. Niben101Scf03628g14021.1 **Mass:** 110528 **Score:** 23 **Expect:** 0.054 **Matches:** 9
sequence match in blast db N.benthamiana Genome v1.0.1 predicted proteins PPC1-L2
Niben101Scf07352g00001.1 **Mass:** 48208 **Score:** 16 **Expect:** 0.31 **Matches:** 4
sequence match in blast db N.benthamiana Genome v1.0.1 predicted proteins PPC3_L5
Niben101Scf03487g00014.1 **Mass:** 49417 **Score:** 15 **Expect:** 0.33 **Matches:** 4
sequence match in blast db N.benthamiana Genome v1.0.1 predicted proteins PPC1-L3

---

4. Niben101Scf00031g00003.1 **Mass:** 110873 **Score:** 20 **Expect:** 0.12 **Matches:** 8
sequence match in blast db N.benthamiana Genome v1.0.1 predicted proteins PPC3-L1

---

5. Niben101Scf25430g00015.1 **Mass:** 110944 **Score:** 8 **Expect:** 1.9 **Matches:** 4
sequence match in blast db N.benthamiana Genome v1.0.1 predicted proteins PPC1-L1

Search Parameters

Type of search : MS/MS Ion Search
Enzyme : Trypsin
Fixed modifications : Carbamidomethyl (C)
Variable modifications : Oxidation (M)
Mass values : Monoisotopic
Protein Mass : Unrestricted
Peptide Mass Tolerance : ± 50 ppm
Fragment Mass Tolerance: ± 0.5 Da
Max Missed Cleavages : 2
Instrument type : Default
Query1 (1003.5218,1+): <no title>
Query2 (1033.5177,1+): <no title>
Query3 (1037.5215,1+): <no title>
Query4 (1054.5266,1+): <no title>
Query5 (1067.5281,1+): <no title>
Query6 (1097.5424,1+): <no title>
Query7 (1111.5348,1+): <no title>
Query8 (1113.5622,1+): <no title>
Query9 (1122.5910,1+): <no title>
Query10 (1140.6165,1+): <no title>
Query11 (1148.5814,1+): <no title>
Query12 (1189.5965,1+): <no title>
Query13 (1204.6193,1+): <no title>
Query14 (1292.6654,1+): <no title>
Query15 (1483.7105,1+): <no title>
Query16 (1511.7617,1+): <no title>
Query17 (1523.7658,1+): <no title>
Query18 (1559.7705,1+): <no title>
Query19 (1579.8317,1+): <no title>

```
Query20 (1658.8160,1+): <no title>
Query21 (1698.8483,1+): <no title>
Query22 (1712.8747,1+): <no title>
Query23 (1765.8030,1+): <no title>
Query24 (1794.8078,1+): <no title>
Query25 (1832.9035,1+): <no title>
Query26 (1878.9783,1+): <no title>
Query27 (1905.9031,1+): <no title>
Query28 (1925.9484,1+): <no title>
Query29 (1946.0054,1+): <no title>
Query30 (2104.0963,1+): <no title>
Query31 (2233.1088,1+): <no title>
Query32 (2249.1061,1+): <no title>
Query33 (2255.0980,1+): <no title>
Query34 (2272.0986,1+): <no title>
Query35 (2285.1684,1+): <no title>
Query36 (2291.2136,1+): <no title>
Query37 (2293.0908,1+): <no title>
Query38 (2305.1709,1+): <no title>
Query39 (2322.1368,1+): <no title>
Query40 (2343.1045,1+): <no title>
Query41 (2364.1440,1+): <no title>
Query42 (2453.2935,1+): <no title>
Query43 (2663.3059,1+): <no title>
Query44 (2677.3077,1+): <no title>
Query45 (3048.4565,1+): <no title>
Query46 (3191.6198,1+): <no title>
Query47 (3323.6885,1+): <no title>
Query48 (3347.5884,1+): <no title>
Query49 (3350.5874,1+): <no title>
Query50 (3460.3783,1+): <no title>
```

Supplementary Fig. S2. Mascot peptide mass fingerprint analysis of the p100 band from tobacco cultured cells extract.

Mascot Search Results

User :
Email :
Search title :
Database : Nicotiana-PEPCs 230719 (11 sequences; 8949 residues)
Timestamp : 3 Oct 2019 at 10:40:00 GMT
Top Score : 19 for Niben101Scf00031g00003.1, sequence match in blast db N.benthamiana Genome v1.0.1 predicted proteins PPC3-L1

Mascot Score Histogram

Protein score is -10*Log(P), where P is the probability that the observed match is a random event.
Protein scores greater than 23 are significant (p<0.05).

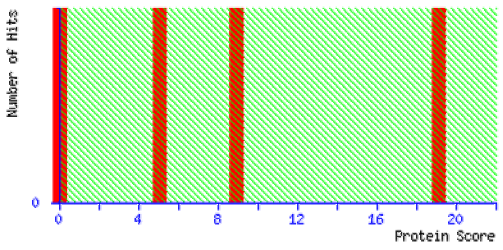

Concise Protein Summary Report

Format As | Concise Protein Summary | Help

Significance threshold p< 0.05 Max. number of hits 20

Re-Search All | Search Unmatched

1. Niben101Scf00031g00003.1 **Mass:** 110873 **Score:** 19 **Expect:** 0.14 **Matches:** 5
sequence match in blast db N.benthamiana Genome v1.0.1 predicted proteins PPC3-L1
Niben101Scf03487g00014.1 **Mass:** 49417 **Score:** 15 **Expect:** 0.38 **Matches:** 3
sequence match in blast db N.benthamiana Genome v1.0.1 predicted proteins PPC1-L3
Niben101Scf03628g14021.1 **Mass:** 110528 **Score:** 8 **Expect:** 1.7 **Matches:** 3
sequence match in blast db N.benthamiana Genome v1.0.1 predicted proteins PPC1-L2
Niben101Scf00212g03010.1 **Mass:** 116969 **Score:** 6 **Expect:** 3 **Matches:** 2
sequence match in blast db N.benthamiana Genome v1.0.1 predicted proteins PPC3-L4
Niben101Scf07352g00001.1 **Mass:** 48208 **Score:** 5 **Expect:** 3.7 **Matches:** 1
sequence match in blast db N.benthamiana Genome v1.0.1 predicted proteins PPC3_L5

---

2. Niben101Scf04036g04008.1 **Mass:** 109934 **Score:** 8 **Expect:** 1.6 **Matches:** 5
sequence match in blast db N.benthamiana Genome v1.0.1 predicted proteins PPC3-L2
Niben101Scf03439g03004.1 **Mass:** 109825 **Score:** 8 **Expect:** 1.6 **Matches:** 5
sequence match in blast db N.benthamiana Genome v1.0.1 predicted proteins PPC3-L3

---

3. Niben101Scf25430g00015.1 **Mass:** 110944 **Score:** 5 **Expect:** 3.2 **Matches:** 2
sequence match in blast db N.benthamiana Genome v1.0.1 predicted proteins PPC1-L1

---

4. Niben101Scf00312g03005.1 **Mass:** 118382 **Score:** 0 **Expect:** 11 **Matches:** 4
sequence match in blast db N.benthamiana Genome v1.0.1 predicted proteins PPC4_L1
Niben101Scf05797g01010.1 **Mass:** 118104 **Score:** 0 **Expect:** 11 **Matches:** 3
sequence match in blast db N.benthamiana Genome v1.0.1 predicted proteins PPC4_L2

Search Parameters

Type of search : Peptide Mass Fingerprint
Enzyme : Trypsin
Fixed modifications : Carbamidomethyl (C)
Variable modifications : Oxidation (M)
Mass values : Monoisotopic
Protein Mass : Unrestricted
Peptide Mass Tolerance : ± 50 ppm
Peptide Charge State : 1+
Max Missed Cleavages : 2
Number of queries : 51
Selected for scoring : 31

Mascot: http://www.matrixscience.com/

Supplementary Fig. S3. Comparison of MALDI fingerprints of the p110 and p100 bands from tobacco cultured cells extract.

Espectros MALDI FINGERPRINT: BANDA1 vs BANDA 2

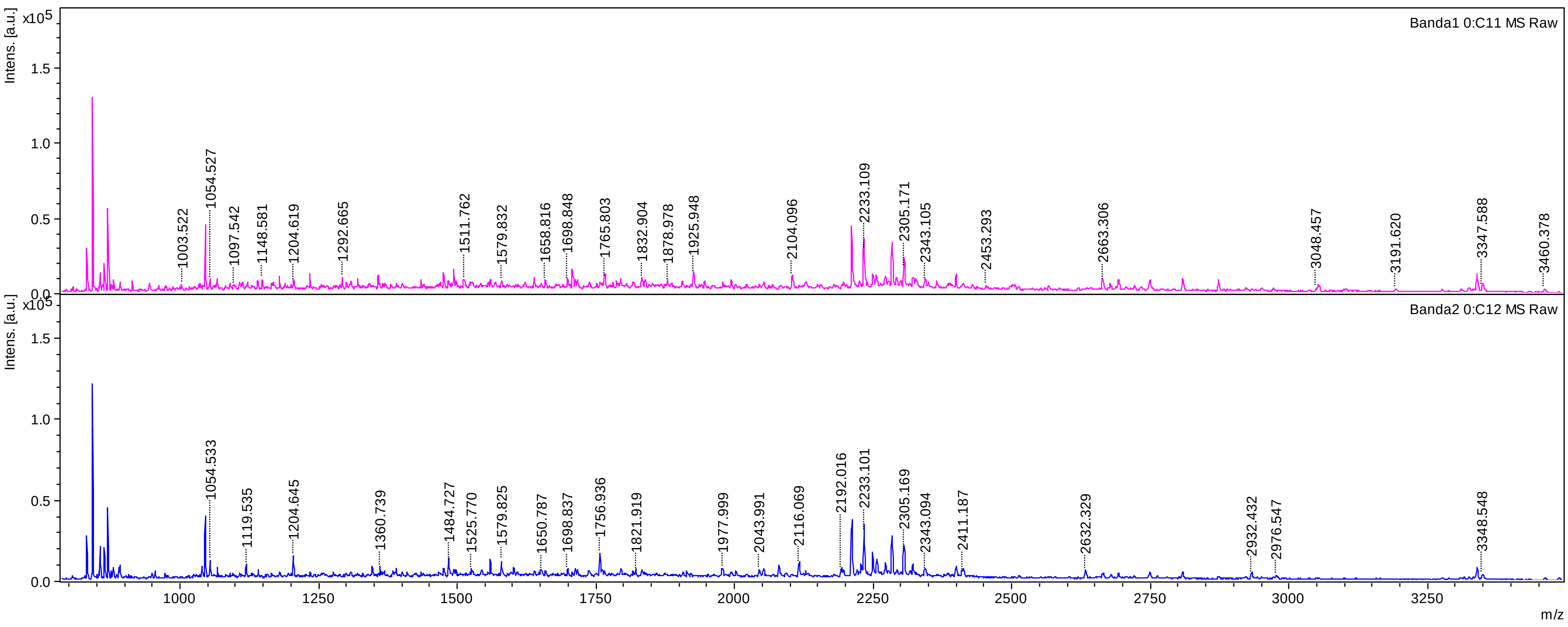

Espectro BANDA 1: Mass List

m/z	m/z
1003.522	1905.903
1033.518	1925.948
1037.521	1946.005
1054.527	2104.096
1067.528	2233.109
1097.542	2249.106
1111.535	2255.098
1113.562	2272.099
1122.591	2285.168
1140.616	2291.214
1148.581	2293.091
1189.596	2305.171
1204.619	2322.137
1292.665	2343.105
1483.711	2364.144
1511.762	2453.293
1523.766	2663.306
1559.771	2677.308
1579.832	3048.457
1658.816	3191.620
1698.848	3323.689
1712.875	3347.588
1765.803	3350.587
1794.808	3460.378
1832.904	
1878.978	

Espectro BANDA 2: Mass List

m/z	m/z
1039.615	1832.854
1054.533	1977.999
1067.533	2043.991
1083.536	2052.009
1119.535	2079.998
1132.554	2116.069
1140.543	2192.016
1195.654	2197.043
1204.645	2228.199
1346.728	2233.101
1360.739	2249.090
1467.713	2259.204
1484.727	2269.121
1525.770	2272.097
1543.765	2293.062
1559.801	2305.169
1579.825	2321.136
1601.825	2343.094
1650.787	2411.187
1698.837	2414.206
1712.855	2632.329
1737.876	2663.305
1756.936	2932.432
1794.820	2976.547
1816.835	3323.636
1821.919	3348.548

Supplementary Fig. S4. Mascot peptide mass fingerprint analysis of the 63 kDa band from the pull down following transient expression of GFP-ATG8CL in Nicotiana benthamiana leaves.

Mascot Search Results

```
User          :
Email         :
Search title  :
Database      : Nicotiana-PEPCs 230719 (11 sequences; 8949 residues)
Timestamp     : 23 Jul 2019 at 10:50:49 GMT
Top Score     : 47 for Niben101Scf03487g00014.1, sequence match in blast db N.benthamiana Genome v1.0.1 predicted proteins PPC1-L3
```

Mascot Score Histogram

Protein score is -10*Log(P), where P is the probability that the observed match is a random event.
Protein scores greater than 23 are significant (p<0.05).

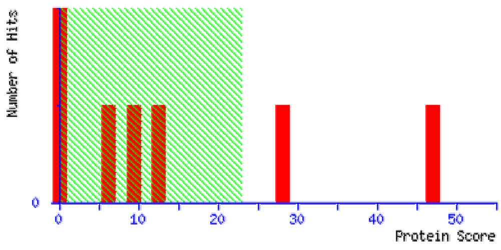

Concise Protein Summary Report

Format As | Concise Protein Summary | Help

Significance threshold p< 0.05 Max. number of hits 20

Re-Search All | Search Unmatched

1. Niben101Scf03487g00014.1 **Mass:** 49417 **Score:** **47** **Expect:** 0.00022 **Matches:** 9
sequence match in blast db N.benthamiana Genome v1.0.1 predicted proteins PPC1-L3

2. Niben101Scf03628g14021.1 **Mass:** 110528 **Score:** **28** **Expect:** 0.019 **Matches:** 10
sequence match in blast db N.benthamiana Genome v1.0.1 predicted proteins PPC1-L2
Niben101Scf07352g00001.1 **Mass:** 48208 **Score:** 0 **Expect:** 11 **Matches:** 1
sequence match in blast db N.benthamiana Genome v1.0.1 predicted proteins PPC3_L5

3. Niben101Scf08191g02001.1 **Mass:** 21846 **Score:** 11 **Expect:** 0.83 **Matches:** 2
sequence match in blast db N.benthamiana Genome v1.0.1 predicted proteins PPC2_L1

4. Niben101Scf00031g00003.1 **Mass:** 110873 **Score:** 11 **Expect:** 0.91 **Matches:** 5
sequence match in blast db N.benthamiana Genome v1.0.1 predicted proteins PPC3-L1

5. Niben101Scf25430g00015.1 **Mass:** 110944 **Score:** 6 **Expect:** 2.5 **Matches:** 4
sequence match in blast db N.benthamiana Genome v1.0.1 predicted proteins PPC1-L1

6. Niben101Scf04036g04008.1 **Mass:** 109934 **Score:** 0 **Expect:** 11 **Matches:** 2
sequence match in blast db N.benthamiana Genome v1.0.1 predicted proteins PPC3-L2

7. Niben101Scf03439g03004.1 **Mass:** 109825 **Score:** 0 **Expect:** 11 **Matches:** 3
sequence match in blast db N.benthamiana Genome v1.0.1 predicted proteins PPC3-L3

Search Parameters

```
Type of search         : Peptide Mass Fingerprint
Enzyme                 : Trypsin
Fixed modifications    : Carbamidomethyl (C)
Variable modifications : Oxidation (M)
Mass values            : Monoisotopic
Protein Mass           : Unrestricted
Peptide Mass Tolerance : ± 50 ppm
Peptide Charge State   : 1+
Max Missed Cleavages   : 2
Number of queries      : 62
Selected for scoring   : 44
```

Mascot: http://www.matrixscience.com/

Supplementary Table S1.

Putative ATG8 interacting motifs (AIMs) in PEPC proteins identified with hfAIM (Xie et al., 2016) and iLIR (Kalvari et al., 2014).

Protein	hfAIM		iLIR		
	Residues	Sequence	Residues	Sequence	PSSM
Arabidopsis thaliana					
AtPPC1	30-36	LVEYDAL	--	--	--
	155-161	EEIFDAL	--	--	--
AtPPC2	30-36	LIEYDAL	--	--	--
	154-160	E**EVFDAL**	155-160	**EVFDAL**	8 (3.9e-01)
	--	--	397-402	AVFTSV	5 (1.0e+00)
AtPPC3	30-36	LVEYDAL	--	--	--
	155-161	EEIFDAL	--	--	--
AtPPC4	122-128	DDIFSQL	--	--	--
	389-395	E**SDWEKI**	390-395	**SDWEKI**	23 (3.2e-03)
	--	--	744-749	PTYLAI	11 (1.5e-01)
Nicotiana benthamiana					
NbPPC1	30-36	LIEYDAL	--	--	--
	--	--	156-161	EVFDAL	8 (3.9e-01)
NbPPC2	29-35	LVEYDAL	--	--	--
	--	--	154-159	EVFDAL	8 (3.9e-01)
NbPPC3.1	30-36	LVEYDAL	--	--	--
	--	--	155-160	EVFDAL	8 (3.9e-01)
NbPPC3.2	30-36	LVEYDAL	--	--	--
	--	--	155-160	EVFDAL	8 (3.9e-01)
NbPPC3.3	30-36	LVEYDAL	--	--	--
	--	--	155-160	EVFDAL	8 (3.9e-01)
NbPPC4	122-128	DDIFNHL	--	--	--
	--	--	445-450	ASFQKL	10 (2e-01)
	--	--	759-764	PTYLAI	11 (1,5e-01)

Highlighted are amino acids identified by both hfAIM and iLIR.
